# Clinical Evaluation of an HTK Solution for Liver Transplantation: A Phase 3 Randomized Pilot Clinical Trial Study

**DOI:** 10.34172/aim.2022.97

**Published:** 2022-09-01

**Authors:** Seyed Ali Malekhosseini, Younes Ghasemi, Javad Rousta, Roghayyeh Aghaei, Sedigheh Kianpour, Manica Negahdaripour, Reza Heidari, Alireza Shamsaeefar, Siavash Gholami, Saman Nikeghbalian

**Affiliations:** ^1^Shiraz Transplant Center, Abu Ali Sina Hospital, Shiraz University of Medical Sciences, Shiraz, Iran; ^2^Pharmaceutical Sciences Research Center, Shiraz University of Medical Sciences, Shiraz, Iran; ^3^Department of Pharmaceutical Biotechnology, School of Pharmacy, Shiraz University of Medical Sciences, Shiraz, Iran

**Keywords:** Clinical trial, HTK, Organ transplantation, Preservation solution, Prognosis, Transplantation

## Abstract

**Background:**

Organ preservation solutions are not easily accessible in Iran, similar to many resource-limited countries. We aimed to evaluate the efficacy of a locally-produced HTK solution among adult liver transplantation candidates in a pilot clinical trial study.

**Methods:**

Adult patients undergoing liver transplantation were randomly allocated into two groups. One received the HTK solution (PharMedCina Inc., Shiraz, Iran), and the second received the commercially available HTK solution (Custodiol^®^).

**Results:**

Overall, 28 individuals entered the study, including 11 and 9 males (78.6% and 64.3%) in the Custodiol^®^ and local HTK groups, respectively. Clinical characteristics, including postoperative biliary complications, reperfusion syndrome, infection and primary non-function (PNF) rates, amount of intraoperative bleeding, length of hospital and ICU stay, peak aspartate aminotransferase (AST) and alanine aminotransferase (ALT), and duration of follow-up were similar between the two groups (*P*>0.05). One patient died in the locally-produced HTK group. The patient underwent re-transplantation 20 days after his first liver transplantation due to PNF. Two patients died in the Custodiol group, both due to PNF of the liver, which occurred five and three days after transplantation. The two groups did not show any difference regarding serum levels of AST, ALT, alkaline phosphatase (ALP), bilirubin, platelet count, prothrombin time and international normalized ratio, white blood cell count, blood urea nitrogen, and creatinine on the first postoperative day and on the day of discharge (*P*>0.05).

**Conclusion:**

Based on the findings of this pilot study with the current sample size, no statistically significant difference was found between our locally-produced HTK solution and Custodiol^®^ regarding clinical outcomes

## Introduction

 According to the World Health Organization (WHO), more than 140 000 organ transplantations are performed each year globally.^1^ Organ transplantation is a complex and delicate practice performed in specific medical centers in the world. A successful organ transplantation depends on multiple factors, among which quality of the organ is one of the leading factors that determine the success of transplantation.^2^

 Organ preservation solutions are among the most important factors that insure the function of an organ after procurement. The solution plays a vital role in ischemia-reperfusion injury and significantly affects primary function of the transplanted organ.^3,4^ Different preservation solutions have been introduced throughout decades, which have been used intermittently in clinical settings. The first was the University of Wisconsin or UW solution, which was first introduced in 1987,^5^ after which the Histidine-Tryptophan-Ketoglutarate (HTK) solution was presented as a potential replacement in 1990. Today, some commonly used and commercially available preservation products include Euro-Collins, UW (Viaspan®), Celsior^®^, Custodiol^®^, and IGL-1^®^.^2^

 The HTK solution has shown some advantages over the UW solution, including lower costs, as well as lower potassium and viscosity, which has made it a more suitable replacement in clinical settings.^6^ Furthermore, studies have shown a lower incidence of biliary complications following HTK usage compared to the UW preservation solution.^7^

 In Iran, despite advancements in surgery and the availability of a national program for transplantation, similar to many resource-limited countries, access to preservation solutions is difficult. Thus, we formulated a local preservation solution, similar in biological structure to the HTK solution.

 In this study, we aimed to evaluate the efficacy of the locally produced HTK solution among a population of adult liver transplantation candidates in a pilot phase 3 clinical trial study.

## Materials and Methods

###  Study Setting

 This study was conducted in the Shiraz Transplant Center affiliated to Shiraz University of Medical Sciences, Shiraz, Iran. Patients undergoing liver transplantation who were above 18 years of age and gave their informed consent to enter the study, were considered for inclusion in this trial.

 Patients with organ donations from outside of the province (in order to limit the cold ischemic time of organs), pregnant or lactating patients, patients with donations after cardiac death, those who were undergoing re-transplantations, and those with machine perfusion, were excluded from the study.

 After entry into the study, patients were randomly allocated in one of the two following groups. The HTK solution (PharMedCina Inc., Shiraz, Iran) was used for organ preservation before transplantation in one group, and the commercially available HTK solution (Custodiol^®^) was utilized in the second group.

###  Randomization

 The eligible study participants (n = 28) were randomized into two study arms (1:1) using the permuted block randomization with a fixed block size of 2. The randomization sequence was generated with an online program available at https://www.sealedenvelope.com/simple-randomiser/v1/lists.

###  Allocation Concealment

 The generated random sequences were inserted in opaque envelops enumerated in sequence from 01 to 28, each of which was used for consecutive study participants.

###  Blinding and Sample Size

- Patients were blinded to the preservation solution used in their liver transplantation.
- In this pilot study, a sample size of 14 individuals was considered for each treatment arm.

###  Primary Outcomes

- Primary non-function (PNF)

###  Secondary Outcomes

- Changes in aspartate aminotransferase (AST) and alanine aminotransferase (ALT) from the first day after transplantation up to the day of discharge from hospital
- Acute rejection during first month after transplantation
- Early patient survival (three months)
- Biliary complications

###  Follow-up

 Patients were followed according to their routine transplantation follow-up schedule. This includes a daily visit for the first week, which changes to visits on every other day during the second post-transplantation week. Doppler sonography is done for patients during the second week. After one month of hospitalization, the patients are sent home and during the following two months, they are visited every two weeks, which then becomes every six months. In case of any complaints or complications, patients are asked to refer to a medical health center and transplantation coordinators are readily available at all times to answer patients’ questions or to schedule a visit in the center for patients.

 Considering the short-term primary and secondary outcomes, the minimum follow-up for each patient included in this study was considered three months.

###  Variables 

 Patients’ data were gathered using a predefined data gathering sheet, which included data on baseline and clinical characteristics. Baseline characteristics including age, sex, body mass index (BMI), and blood type, clinical characteristics including underlying disease, duration of liver disease prior to transplantation, cause of liver failure, and Model for End-Stage Liver Disease (MELD) score, surgery related data including type of organ transplantation (deceased or living organ donation), type of hepatectomy used, and warm and cold ischemic time, clinical outcomes including rate of reperfusion syndrome, postoperative complications, in-hospital rejection, PNF, amount of bleeding during surgery, length of hospital and ICU stay, and mortality were documented for each individual. Furthermore, data on the patients’ laboratory tests were registered on consecutive days from the day of transplantation to discharge in order to document changes. Donor related data including age and sex were also gathered for each patient.

###  Statistical Analysis

 Data was analyzed using the Statistical Software for Social Sciences (SPSS Inc., Chicago, IL, USA), for Windows, version 20. The intention-to-treat protocol was used for data analysis. Qualitative data were compared between the two groups using the χ2 test or the Fisher’s exact test, when necessary. Quantitative data with a normal distribution were compared between the groups using the independent *t *test and variables without a normal distribution were compared using the Mann-Whitney U test. For comparison of survival between the two groups, the Kaplan-Meier plot and the log-rank test were used.

## Results

 In total, 28 individuals entered the study, including 11 males (78.6%) in the Custodiol HTK group and 9 males (64.3%) in the local HTK group. Mean (SD) age in the local and Custodiol HTK groups was 46.2 ± 13.9 years and 48.0 ± 8.1 years, respectively. Overall, the most common causes of liver failure in the study groups were non-alcoholic steatohepatitis (NASH) (7/28), PSC (4/28), autoimmune hepatitis (3/28), and hepatitis B virus (HBV) (3/28). The two groups did not show any difference in baseline clinical characteristics. The baseline and clinical characteristics of patients are shown in Tables 1 and 2.

**Table 1:** Baseline Characteristics of the Study Population

**Characteristics**	**CustodiolHTK (n=14)**	**Local HTK (n=14)**	**Mean Difference (95% CI)**	**Effect Size (95% CI)**	***P*** ** Value**
Age, years	48.0 ± 8.1	46.2 ± 13.9	1.7 (-7.1–10.5)	0.15 (-0.59–0.89)	0.694
Sex, No. (%)					
Male	11 (78.6)	9 (64.3)	—	—	0.678
Female	3 (21.4)	5 (35.7)	—	—	
BMI, kg/m^2^	24.9 ± 2.3	24.9 ± 3.3	-0.007 (-2.2–2.2)	-0.002 (-0.74–0.73)	0.995
Blood type, No. (%)					
O	8 (57.1)	8 (57.1)	—	—	0.900
A	3 (21.4)	3 (21.4)	—	—	
B	2 (14.3)	2 (14.3)	—	—	
AB	1 (7.1)	1 (7.1)	—	—	
Underlying disease, No. (%)					
Cardiac	1 (7.1)	0	—	—	0.633
Diabetes	2 (14.3)	4 (28.6)	—	—	
Renal failure	1 (7.1)	1 (7.1)	—	—	
Duration of liver disease, months	55.1 ± 46.5	54.4 ± 64.2	0.6 (-51.1–52.3)	0.01 (-0.84–0.86)	0.979
Cause of liver failure, No. (%)					
Nonalcoholic steatohepatitis	3 (21.4)	4 (28.6)	—	—	0.464
Primary sclerosing cholangitis	4 (28.5)	0	—	—	
Cryptogenic	0	2 (14.3)	—	—	
Autoimmune hepatitis	2 (14.3)	1 (7.1)	—	—	
Hepatitis C virus	1 (7.1)	0	—	—	
Alcoholic hepatitis	0	1 (7.1)	—	—	
Budd Chiari	0	1 (7.1)	—	—	
Hepatitis B virus	2 (14.3)	1 (7.1)	—	—	
Hepatitis B virus + Hepatocellular carcinoma	1 (7.1)	0	—	—	
Hyperoxalemia	0	1 (7.1)	—	—	
Primary biliary cirrhosis	1 (7.1)	1 (7.1)	—	—	
Primary sclerosing cholangitis + Hepatocellular carcinoma	0	1 (7.1)	—	—	
Wilson's disease	0	1 (7.1)	—	—	
MELD score	18.3 ± 5.1	17.1 ± 3.9	1.1 (-2.5–4.9)	0.25 (-0.53–1.04)	0.525
Type of organ transplantation, No. (%)					
Deceased	13 (92.9)	10 (71.4)	—	—	0.326
Living	1 (7.1)	4 (28.6)	—	—	
Type of hepatectomy, No. (%)					
Piggyback	11 (78.6)	12 (85.7)	—	—	0.622
Standard	3 (21.4)	2 (14.3)	—	—	
Warm ischemic time; minutes	36.2 ± 10.2	36.9 ± 5.2	-4.4 (-14.4–5.4)	-0.08 (-0.82–0.65)	0.818
Cold ischemic time; minutes	243.5 ± 190.3	197.5 ± 183.3	214.2 (-143.3–51.9)	0.24 (-0.50–0.98)	0.520

BMI, body mass index; MELD, model for end-stage liver disease. All plus-minus are means and standard deviation unless stated otherwise.

**Table 2:** Clinical Characteristics and Outcomes of Patients

**Characteristics**	**CustodiolHTK (n=14)**	**Local HTK (n=14)**	**Mean Difference (95% CI)**	**Effect Size (95% CI)**	***P *** **Value**
Reperfusion syndrome, No. (%)					
Yes	7 (50)	5 (41.7)	—	—	0.671
No	7 (50)	7 (58.3)	—	—	
Postoperative complications, No. (%)					
Bleeding	0	2 (14.3)	—	—	0.219
Bleeding + portal thrombosis	1 (7.1)	0	—	—	
Infections, No. (%)					
None	8 (57.1)	11 (78.6)	—	—	0.483
Site of surgery	0	1 (7.1)	—	—	
Cytomegalovirus	1 (7.1)	0	—	—	
Pneumonia	1 (7.1)	1 (7.1)	—	—	
Urinary	3 (21.4)	1 (7.1)	—	—	
Sepsis	1 (.1)	0	—	—	
In-hospital rejection, No. (%)					
Yes	2 (14.3)	5 (35.7)	—	—	0.385
No	12 (85.7)	9 (64.3)	—	—	
Primary non-function, No. (%)					
Yes	2 (14.3)	1 (7.1)	—	—	0.541
No	12 (85.)	13 (92.9)	—	—	
Biliary complications during hospitalization; n (%)					
Biloma	0	1 (7.1)	—	—	0.365
Stricture	1 (7.1)	0	—	—	
No	13 (92.9)	13 (92.9)	—	—	
Amount of bleeding, cc	2084 ± 987	1700 ± 1813	384 (-809–1579)	0.26 (-0.52–1.05)	0.512
ICU stay, days	10 ± 4.2	7.9 ± 2.7	2.0 (-0.7–4.9)	0.58 (-0.19–1.34)	0.145
Hospital stay, days	12.1 ± 5.5	14.0 ± 3.2	-1.8 (-5.4–1.7)	-0.40 (-1.16–0.36)	0.303
Peak aspartate aminotransferase, IU/L	1214 ± 694	1101 ± 1175	113 (-859–1085)	0.11 (-0.74–1.01)	0.799
Peak alanine transaminase, IU/L	899 ± 499	682 ± 599	21 (-314–49)	0.39 (-0.52–1.30)	0.401
Duration of follow-up, days	344 ± 166	265 ± 142	79 (-40–200)	-0.61 (-1.37–0.14)	0.185
Mortality, No. (%)					
Yes	2 (14.3)	1 (7)	—	—	0.541
No	12 (85.7)	13 (93)	—	—	

ICU, Intensive care unit. All plus-minus are means and standard deviation unless stated otherwise.

 The two groups were similar regarding clinical characteristics including postoperative biliary complications, reperfusion syndrome, infection rates, PNF rates, amount of intraoperative bleeding, length of hospital and ICU stay, peak AST and ALT, and duration of follow-up (*P* > 0.05).

**Figure 1:**
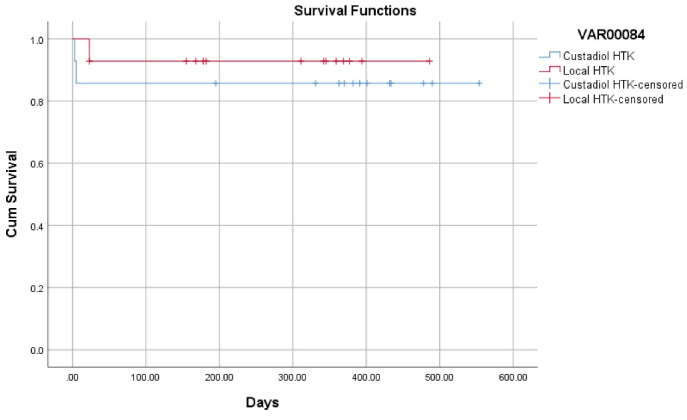
Kaplan Kaplan-Meier Plot for Survival in the Two Comparison Groups

 One patient died in the locally produced HTK group. The patient had a re-transplantation 20 days after his first liver transplantation due to PNF, and the patient died three days after his re-transplantation due to another PNF of the liver. For the re-transplantation, the patient received the Custodiol HTK solution. Two patients died in the Custodiol HTK group, both due to PNF of the liver, which occurred five and three days after transplantation. One of the patients underwent re-transplantation on the fifth day of his primary transplantation and died on the same day (survival rate 92.9% for the local HTK group vs. 85.7% for the Custodiol HTK group; *P* = 0.520) (Figure 1).

 Regarding laboratory tests on consecutive days after receiving the HTK solutions, the two groups did not show any difference in serum levels of AST, ALT, alkaline phosphatase (ALP), bilirubin, platelet count, prothrombin time and international normalized ratio, white blood cell count, blood urea nitrogen, and creatinine on the first postoperative day and on the day of discharge (*P* > 0.05) (Table 3).

**Table 3:** Laboratory Tests on Postoperative Days

**Variables**		**CustodiolHTK (n=14)**	**Local HTK (n=14)**	**Mean Difference (95% CI)**	**Effect Size (95% CI)**	***P*** ** Value**
Total bilirubin - mg/dL	Day 1	6.8 ± 5.0	4.4 ± 6.5	2.4 (-2.0–7.0)	0.42 (-0.33–1.16)	0.273
	Day 7	3.1 ± 2.7	1.0 ± 0.5	2.0 (0.49–3.6)	1.09 (0.23–1.92)	0.012
	On discharge	2.0 ± 1.4	1.6 ± 3.3	0.4 (-1.7–2.5)	0.16 (-0.61–0.93)	0.682
AST (IU/L)	Day 1	1506 ± 1539	999 ± 1019	507 (-506–1521)	0.38 (-0.36–1.13)	0.313
	Day 7	71.4 ± 74.1	65.1 ± 25.1	6.3 (-39.0–51.6)	0.11 (-0.68–0.92)	0.776
	On discharge	41.9 ± 30.9	32.3 ± 16.3	9.5 (-10.9–30.0)	0.39 (-0.42–1.20)	0.345
ALT (IU/L)	Day 1	929 ± 644	630 ± 519	298 (-163–760)	0.51 (-0.26–1.2)	0.196
	Day 7	228 ± 188	180 ± 115	47 (-81–177)	0.31 (-0.49–1.11)	0.454
	On discharge	121 ± 5	113 ± 66	8 (-50–66)	0.11 (-0.67–0.90)	0.771
ALP (IU/L)	Day 1	414 ± 487	365 ± 321	49 (-275–374)	0.12 (-0.63–0.87)	0.757
	Day 7	448 ± 295	423 ± 258	25 (-208–259)	0.09 (-0.71–0.89)	0.824
	On discharge	470 ± 348	440 ± 389	-29 (-280–340)	0.08 (-0.71–0.90)	0.844
PT (s)	Day 1	29.3 ± 13.2	24.1 ± 9.2	5.2 (-3.7–14.1)	0.46 (-0.30–1.22)	0.242
	Day 7	15.0 ± 2.4	13.7 ± 1.5	1.2 (-1.2–3.7)	0.58 (-0.50–1.65)	0.299
	On discharge	14.7 ± 1.9	15.8 ± 5.8	-1.1 (-8.3–6.0)	-0.26 (-1.45–0.93)	0.669
INR	Day 1	2.6 ± 1.1	1.9 ± 0.4	0.6 (-0.006-1.3)	0.78 (-0.006–1.56)	0.052
	Day 7	1.2 ± 0.2	1.1 ± 0.1	0.1 (-0.08–0.3)	-0.72 (-0.38–1.80)	0.206
	On discharge	1.1 ± 0.1	1.2 ± 0.4	-0.09 (-0.5–0.3)	-0.28 (-1.46–0.92)	0.654
White blood cell count	Day 1	12230 ± 4993	12007 ± 4451	-776 (-4520–2967)	-0.16 (-0.91–0.59)	0.673
	Day 7	8845 ± 4093	8108 ± 4590	737 (-2976–4451)	0.16 (-0.63–0.9)	0.685
	On discharge	8263 ± 2971	8314 ± 4448	-51 (-3281–3179)	-0.01 (-0.80–0.77)	0.974
Platelet count (per microliter)	Day 1	96384 ± 113672	77214 ± 59732	19.170 (-55115–93455)	0.21 (-0.54–0.96)	0.584
	Day 7	93000 ± 69951	97538 ± 63481	-4538 (-6159–52682)	-0.06 (-0.87–0.73)	0.869
	On discharge	138363 ± 62944	181500 ± 121521	-43136 (-132444–4611)	-0.37 (-1.16–0.42)	0.361
BUN level (mg/dL)	Day 1	20.2 ± 9.7	28 ± 13.9	-7.7 (-17.2–1.7)	-0.64 (-1.41–0.13)	0.108
Creatinine level (mg/dL)	Day 1	1.1 ± 0.6	1.5 ± 1.0	-0.3 (-1.0–0.3)	0.17 (-0.66–1.01)	0.278

AST, Aspartate aminotransferase; ALP, alkaline phosphatase; PT, prothrombin time; INR, International normalized ratio; BUN, blood urea nitrogen. All plus-minus are means and standard deviation unless stated otherwise.

 The mean donor age in the local and Custodiol HTK groups was 41.9 ± 18.3 years and 44.8 ± 18.7 years (mean difference: 2.9, 95% CI = -11.1–17.0), respectively. Donor specifics are presented in Table 4.

**Table 4:** Donor Related Characteristics Among the Study Population

**Variables**	**Custodiol HTK (n=14)**	**Local HTK (n=14)**	**Mean Difference (95% CI)**	***P*** ** Value**
Age (years)	44.8 ± 18.7	41.9 ± 18.3	2.9 (-11.1–17.0)	0.673
**Sex; n (%)**				
Male	10 (69.2)	13 (92.9)	-	0.326
Female	4 (23.1)	1 (7.1)	-	

## Discussion

 In this study, a generic locally produced HTK solution was evaluated and its clinical efficacy was compared to that of a commercially available and well-established standard Custodiol HTK solution in a clinical trial. Accordingly, the two solutions were found to be similar regarding clinical outcomes among adult patients undergoing liver transplantation.

 In literature, HTK solutions have been mostly compared to UW preservation products with regard to clinical outcomes after transplantation in the settings of clinical trial studies,^8,9^ and some have focused on developing new formulations and preservation solutions.^10-12^

 The effect of preservation solutions on the organ is mainly considered to be short-term. One of the most important aspects to consider with preservations solutions in clinical settings is the post-transplantation biliary complications. One mechanism through which preservation solutions are believed to contribute to the occurrence of biliary complications is the role of the solution in better preservation of the bile ducts, which consequently leads to biliary complications. One report concluded that the preservation solution used for organ preservation even plays a more important role than the cold ischemic time in prevention of postoperative biliary stricture.^13^

 The rate of PNF after using HTK solutions in liver transplantation has been variable in different clinical trial studies depending on the study sample and design^14-20^ and graft dysfunction has been reported to be up to 27% in literature.^21^ More specifically, occurrence of organ dysfunction is believed to be dependent on multiple factors including ischemia time, quality of the organ (lower with marginal organs), immunological matching, and graft reperfusion, which includes the type of preservation solution.^21^ Literature has shown that occurrence of PNF is considered to be unrelated to any factors related to surgery and is highly dependent on the organ preservation method used.^22^ In our study, two patients (14.2%) in the local HTK group and one patient (7.1%) in our Custodiol HTK group developed PNF. Occurrence of PNF is a relatively rare entity itself, and according to an unpublished report, the rate of PNF among adult liver transplantation recipients in our center is between 5 to 11%.

 Different studies have reported variable one-year survival rates among patient with the use of the HTK preservation products. The overall one-year survival rate in our center among adult liver transplantation recipients is reported to be 86.6% (unpublished data). In this randomized clinical trial, we had two patients (14.2%) who died during our follow-up period within the local HTK group (mean follow-up duration of 344 days), which is similar to our documented survival rates among adult liver recipients. In the group that received our locally produced HTK solution, one patient died due to PNF. The patient received the locally produced HTK during his primary transplantation and received the Custodiol^®^ HTK solution at re-transplantation. The disparity in death rates between the two groups was primarily due to the short follow-up and study design. However, considering the main goal of the study, we did not record any difference in primary and secondary clinical outcomes between the commercially available HTK solution and our locally produced solution.

 One of the most important factors believed to affect patient outcomes are donor characteristics. In our study, donor baseline characteristics and type of organ donation (deceased or living) were similar between the comparison groups. The cold and warm ischemic times were similar in both groups, minimizing any bias related to organ selection and transplantation between the two comparison groups.

 Since the first preservation solution was introduced, other countries and institutions have developed different formulations of preservation solutions for use in organ transplantation.^10-12,22^ Our locally produced HTK product is much cheaper and more available (for our region) than the commercially available HTK solutions. Considering the existing limitations in facilities in our region, if these results are confirmed in larger studies, this solution can be used as a substitute for the existing HTK products. This is especially important in middle- and low-income countries that have limited access to preservation solutions.

 This study was not without limitations. Firstly, due to limitation in resources, we were not able to produce a large quantity of the local HTK fluid; as a result, this study was conducted as a pilot study and a larger study in the future will establish the exact efficacy and side effects of the locally produced HTK solution. Accordingly, other methods of statistical analysis would help to definitely conclude if the two products are equivalent. This study was primarily conducted in an adult population, and future studies should also be conducted among pediatric patients undergoing liver transplantations. Considering the short-term effects of the preservation solution on the organ of transplantation and the patient, we only had short-term outcomes set as our primary and secondary goals. The locally produced HTK solution was only evaluated for liver transplantations and other organs will be studied in the future. To the best of the authors’ knowledge, this is the first locally produced preservation product for organ transplantation in Iran.

 In conclusion, based on the obtained results with the current sample size in this pilot study, no statistically significant difference was found between our locally produced HTK solution and the commercially available HTK product (Custodiol^®^) regarding clinical outcomes.
